# Supplementary material for: Stem cell homeostasis regulated by hierarchy and neutral competition
Source: Commun Biol. 2022 Nov 18;5:1268. doi: 10.1038/s42003-022-04218-7 (PMC9674595; doi:10.1038/s42003-022-04218-7)
Supplement: Supplementary file 2 — Supplementary Information [file 42003_2022_4218_MOESM2_ESM.pdf]

Supplementary Information

For

Stem cell homeostasis regulated by hierarchy and neutral competition

Asahi Nakamuta, Kana Yoshido, and Honda Naoki

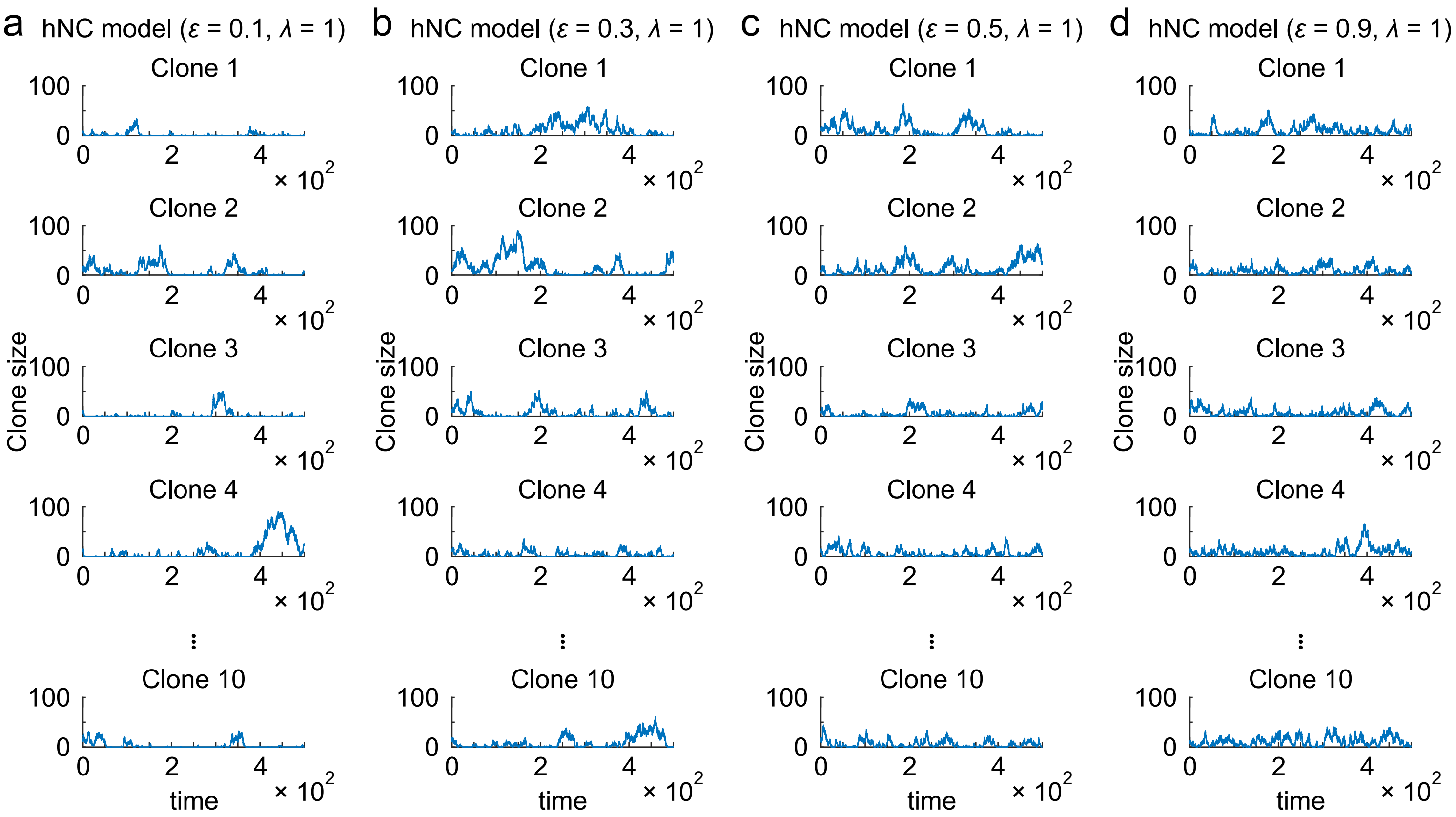

Supplementary Figure 1: Time-series of clone sizes in the hNC model under various conditions. Time-series of clone sizes in the hNC model with different proliferation rates of master stem cells, ε. The simulation included 10 types of clones in 100 competitive stem cells in the open layer, in which the clone size was initially uniform, that is $n_k = 10$.

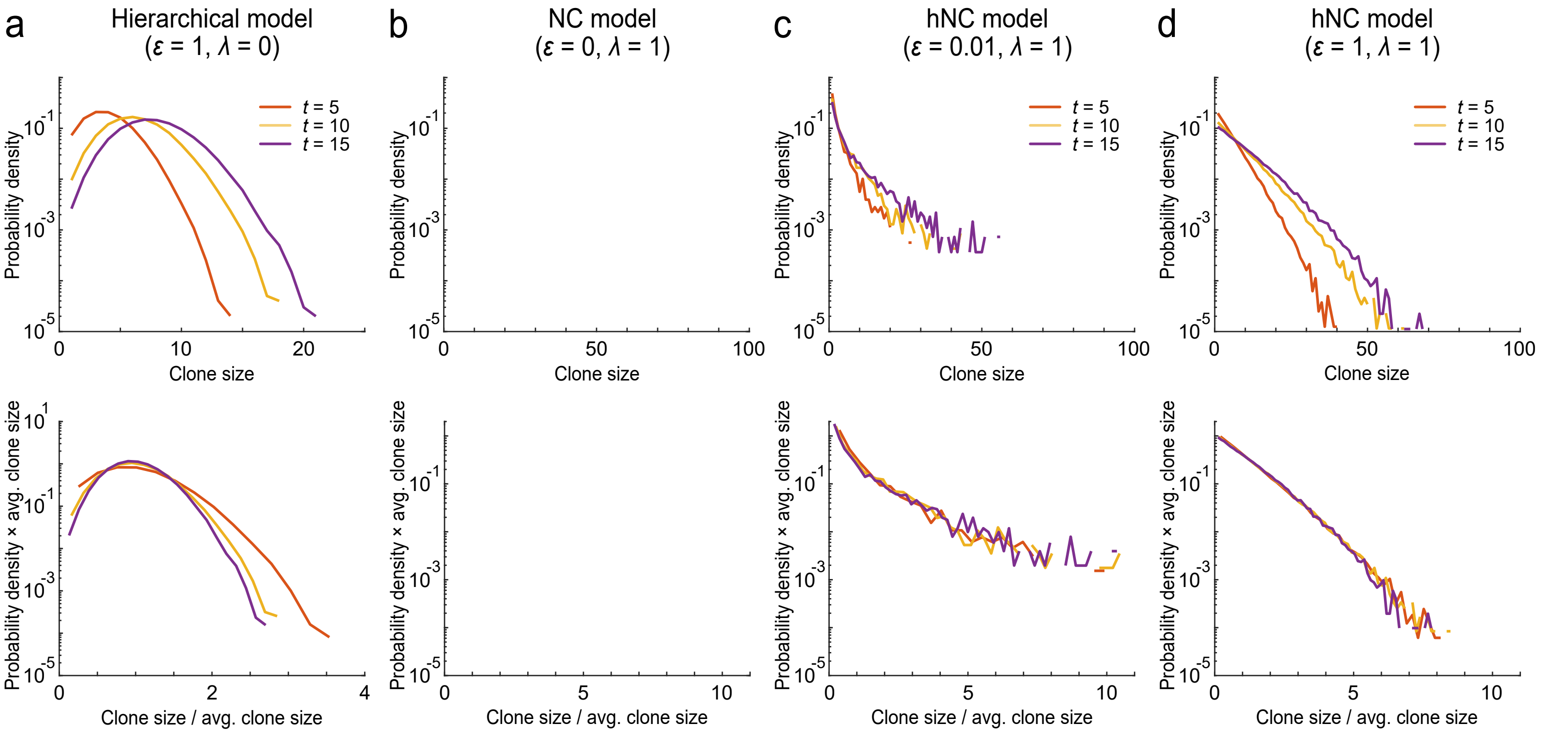

Supplementary Figure 2: Scaling law of clone size distribution in the NC and hNC models with labelling master stem cells.

Probability distribution of the population size of pulse-labeled clones. Simulations were performed 100,000 times using 10 master stem cells and 100 competitive stem cells. One of the master stem cells was randomly labeled. The labeled clonal size distributions are plotted at different time points in the (a) hierarchical model, (b) NC model, and (c, d) hNC model under the two conditions. The clone size distribution is not shown in the NC model, because the NC model has no master stem cells. The upper and lower panels show the distributions before and after scaling by the average clone size at each time point, respectively, as shown in equation [1].

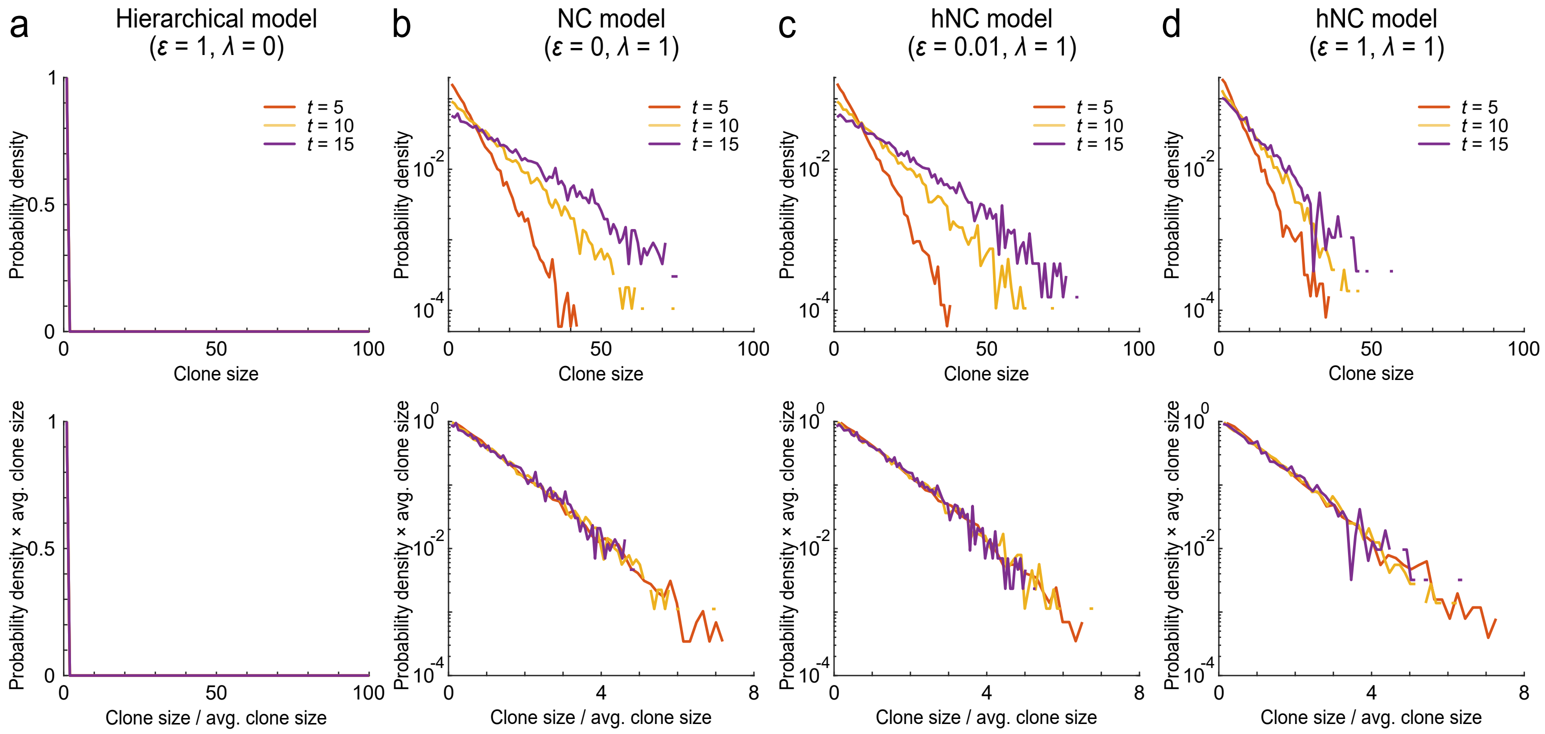

Supplementary Figure 3: Scaling law of clone size distribution in the NC and hNC models with labeling of stem cells in the open layer.

Probability distribution of the population size of pulse-labeled clones. Simulations were performed 100,000 times using 10 master stem cells and 100 competitive stem cells. One of the stem cells in the open layer was randomly labeled: non-master stem cells in the hierarchical model and competitive stem cells in the NC and hNC models. The labeled clonal size distributions are plotted at different time points in the (a) hierarchical model, (b) NC model, and (c, d) hNC model under the two conditions. Upper and lower panels show the distributions before and after scaling by the average clone size at each time point, respectively, as shown in equation [1].

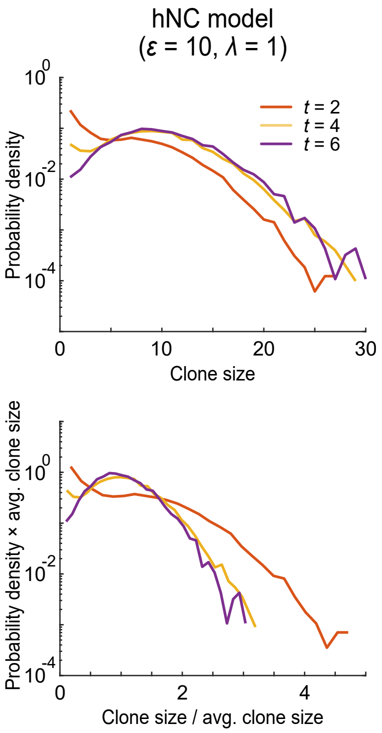

Supplementary Figure 4: Scaling law of clone size distribution not satisfied in the hNC model with high-cycling master stem cells.

Probability distribution of the population size of pulse-labeled clones under the condition where the proliferation of master stem cells is much larger than that of competitive stem cells. Simulations were performed 100,000 times using 10 master stem cells and 100 competitive stem cells. One of the master and competitive stem cells was randomly labeled. The labeled clonal size distributions are plotted at different time points in the hNC model, with master stem cells being much more active than competitive stem cells. Upper and lower panels show the distributions before and after scaling, respectively, as shown in equation [1].

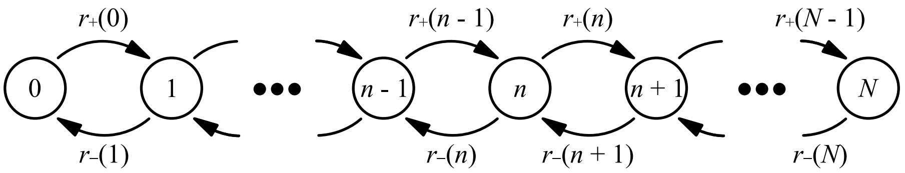

Supplementary Figure 5: State transition diagram of clonal expansion.

Clonal size stochastically changes because of neutral competition and supply from a master stem cell. n and N indicate the size of a clone of interest and the total number of competitive stem cells, respectively. $r_+(n)$ and $r_-(n)$ denote the transition probabilities that the clone size increases and decreases by one from n, respectively.
